# Mean platelet volume: a novel predictive marker for mortality in patients with acute mesenteric ischemia?

**DOI:** 10.1186/1749-7922-9-30

**Published:** 2014-04-15

**Authors:** Ergenekon Karagöz, Alexis Kofi Okoh, Alpaslan Tanoglu

**Affiliations:** 1 Department of Infectious Diseases and Clinical Microbiology, GATA Haydarpasa Training Hospital, 34668, Uskudar Istanbul, Turkey; 2 Cleveland Clinic Foundation, Center for Endocrine Surgery, 9500 Euclid Ave, Cleveland, OH 44195, USA; 3 Department of Gastroenterology, GATA Haydarpasa Training Hospital, Istanbul, Turkey

## 

Dear Editor,

We read the article entitled “Mean Platelet Volume as a potential prognostic marker in patients with acute mesenteric ischemia (AMI)-retrospective study” by Altintoprak et al with interest [1]. The authors concluded that Mean Platelet Volume (MPV) values at presentation were higher among non-survivors than survivors and might be beneficial in predicting patients with poor prognosis and in the planning of re-operations. The ready availability of this parameter at no additional cost may encourage its utilization in clinical practice. To the best of our knowledge, this is the first study investigating the relationship between MPV and AMI. We would like to thank to the authors for their valuable contribution. On the other hand, we would like to report a few concerns regarding this study from a methodological point of view.

Firstly, the prognosis of AMI is related to late diagnosis, sepsis and colonic involvement [2]. Early evaluation in high-risk patients and resection of necrosed intestinal segments as soon as possible prior to sepsis may reduce the hospital mortality rate [2]. In this context, the authors could have compared and evaluated their cases according to these parameters that affect disease severity.

Secondly, previous studies have demonstrated that diabetes mellitus, peripheral artery disease, acute coronary syndromes, autoimmune disorders, thrombocytopenia, congestive heart failure, acute pulmonary emboli, thyroid functional abnormalities, local or systemic infections, malignancy, inflammatory diseases, and many drugs may potentially affect MPV levels [3]. Although, the authors only described the presence of arteriosclerosis related conditions in their patients, it would have been better, if the authors had mentioned these other MPV effecting factors while assessing the associations between MPV and AMI.

Additionally, the authors did not mention about the type of the tube (ethylenediaminetetraacetic acid (EDTA) or citrate tube) in which blood tests were performed. As reported earlier on in previous studies, MPV levels increase over time in EDTA anti coagulated samples [4,5]. So, it would have been relevant, if the authors had specified how much time elapsed between taking the blood samples and measuring MPV because a delay in measurements may affect the MPV values [6].

We believe that the findings of Altintoprak et al will lead to further research concerning the relationship between MPV and AMI [1]. Nevertheless, it should be kept in mind that MPV alone without other inflammatory markers (e.g. C-reactive protein, sedimentation rate) may not provide certain information about the inflammatory status of the patient. Therefore, we are of the opinion that MPV should be accompanied by other serum inflammatory markers.

## Competing interests

The authors declare that they have no competing interests.
